# Disparities in rate, triggers, and management in pediatric and adult cases of suspected drug‐induced anaphylaxis in Canada

**DOI:** 10.1002/iid3.201

**Published:** 2017-11-01

**Authors:** Sofianne Gabrielli, Ann E. Clarke, Harley Eisman, Judy Morris, Lawrence Joseph, Sebastien La Vieille, Peter Small, Rodrick Lim, Paul Enarson, Michal Zelcer, Edmond S. Chan, Chris Mill, Moshe Ben‐Shoshan

**Affiliations:** ^1^ Division of Pediatric Allergy and Clinical Immunology, Department of Pediatrics McGill University Health Centre Montreal Quebec Canada; ^2^ Division of Rheumatology, Department of Medicine, Cumming School of Medicine University of Calgary Calgary Alberta Canada; ^3^ Department of Emergency Medicine Montreal Children's Hospital McGill University Health Centre Montreal Quebec Canada; ^4^ Department of Emergency Medicine Hôpital du Sacré‐Coeur Montreal Quebec Canada; ^5^ Department of Epidemiology and Biostatistics McGill University Montreal Quebec Canada; ^6^ Food Directorate Health Canada Ottawa Ontario Canada; ^7^ Département sciences des aliments, Faculté des sciences de l'agriculture et de l'alimentation Université Laval Québec City Québec Canada; ^8^ Division of Allergy and Clinical Immunology Jewish General Hospital, McGill University Montreal Quebec Canada; ^9^ Department of Paediatrics and Emergency Medicine Children's Hospital at London Health Science Centre London Ontario Canada; ^10^ Division of Emergency Medicine Department of Pediatrics University of British Columbia Vancouver British Columbia Canada; ^11^ Division of Allergy and Immunology Department of Pediatrics BC Children's Hospital University of British Columbia Vancouver British Columbia Canada

**Keywords:** Anaphylaxis, anti‐bacterial agents, anti‐inflammatory agents (non‐steroidal), drug hypersensitivity, skin tests

## Abstract

**Introduction:**

Data is sparse on drug‐induced anaphylaxis (DIA) and there have not been studies assessing the differences in clinical characteristics and management of DIA between adults and children.

**Objective:**

We assessed the percentage, diagnosis, and management of DIA among all anaphylaxis visits in three pediatric and one adult emergency departments (ED) across Canada.

**Methods:**

Children presenting to the Montreal Children's Hospital (MCH), British Columbia Children's Hospital (BCCH), and Children's Hospital at London Health Sciences Center and adults presenting to Hôpital du Sacré‐Coeur with anaphylaxis were recruited as part of the Cross‐Canada Anaphylaxis Registry. A standardized data form documenting the reaction and management was completed and patients were followed annually to determine assessment by allergist and use of confirmatory tests.

**Results:**

From June 2012 to May 2016, 51 children were recruited from the pediatric centers and 64 adults from the adult center with drug‐induced anaphyalxis. More than half the cases were prospectively recruited. The percentage of DIA among all cases of anaphylaxis was similar in all three pediatric centers but higher in the adult center in Montreal. Most reactions in children were triggered by non‐antibiotic drugs, and in adults, by antibiotics. The majority of adults and a third of children did not see an allergist after the initial reaction. In those that did see an allergist, diagnosis was established by either a skin test or an oral challenge in less than 20% of cases.

**Conclusions:**

Our results reveal disparities in rate, culprit, and management of DIA in children versus adults. Further, most cases of suspected drug allergy are not appropriately diagnosed. Guidelines to improve assessment and diagnosis of DIA are required.

## Introduction

Drug‐induced anaphylaxis (DIA) is a life‐threatening allergic reaction involving at least two organ systems and/or hypotension triggered by a drug exposure 1, 2. Studies report that eight out of one million people will have DIA yearly 3, and that 1 case per 4000 Emergency Department (ED) visits will be due to DIA 4. A recent study conducted in Australia found that hospital admission rates due to DIA have increased by 6.8% per year over 16 years and that DIA was the leading cause of fatal anaphylaxis 5. In the United States, drugs were also found to be the most common cause of fatal anaphylaxis with fatalities significantly increasing from 1999 to 2010 6.

Currently there are no prospective studies assessing the clinical characteristics and management of DIA. Furthermore, no studies so far have assessed differences in clinical characteristics and management of DIA between pediatric and adult EDs. We assessed the percentage, demographics, clinical characteristics, and management including the use of confirmatory tests to diagnose DIA cases treated in three pediatric EDs and one adult ED across Canada.

## Methods

### Study design

From June 2012 to May 2016, children presenting to the Montreal Children's Hospital (MCH) ED and adults presenting to the Hôpital du Sacré‐Coeur (HSC) EDs with anaphylaxis were recruited as part of the Cross‐Canada Anaphylaxis Registry (C‐CARE). Over a 2‐year period, from June 2014 to May 2016, children presenting to the British Columbia Children's Hospital (BCCH) and Children's Hospital at London Health Sciences Centre (LHSC) EDs with anaphylaxis were recruited for C‐CARE. The MCH and HSC are tertiary hospitals located in Montreal, Quebec that treat approximately 80,000 and 60,000 patients annually in their EDs, respectively. The BCCH is a tertiary pediatric center located in Vancouver, British Columbia that treats approximately 45,000 patients annually in their ED. The LHSC is a teaching hospital located in London, Ontario, treating 36,000 patients annually in their ED.

This study followed the RECORD guideline for observational studies. Data on patients were collected either prospectively or retrospectively. Prospective data was collected at the time of patient presentation. The treating physician identified cases of anaphylaxis and with the help of a trained research member obtained consent and completed a standardized data entry form documenting symptoms, triggers, and management of anaphylaxis. Data on missed cases that were not recruited at the time of presentation to the ED was collected retrospectively. In brief, all cases presenting to the ED were reviewed according to ICD‐10 codes related to allergic reactions/anaphylaxis based on a previously validated algorithm 7, 8_._ Anaphylaxis was defined as the involvement of two or more organ systems after exposure to a possible allergen or hypotension after exposure to a known allergen 9. Only prospective and retrospective cases meeting the definition of anaphylaxis as determined by two independent reviewers (SG and MBS) were included. Consenting prospective patients or families (in the case of children) were contacted annually to determine if they had been seen by an allergist and if the culprit drug was confirmed through the use of skin tests or an oral challenge. Treating allergists were contacted and asked to provide documented results of skin tests and challenges. Data regarding the use of confirmatory tests for retrospective cases was obtained through chart review for patients who had been seen at the study centers. The study was approved by the McGill University Ethics Committee, the Research Ethics Board of the Hôpital du Sacré‐Coeur, the University of British Columbia/Children's, and Women's Health Center of British Columbia Research Ethics Board and Health Science Research Ethics Board at Western University.

### Statistical analysis

All statistical analyses were done using R version 3.2.2. (R Core Team [2013]; R: A language and environment for statistical computing; R Foundation for Statistical Computing, Vienna, Austria). Percentages with a 95% confidence intervals (CI, binomial or multinomial for variables with more than two categories), were used to assess patient demographics, symptoms, culprit drugs, reaction severity, management, and percentage of DIA cases. Univariate and multivariate logistic regression models were compared to estimate factors associated with reaction severity, assessment by an allergist, and established drug allergy for the pediatric and adult EDs. All variables, excluding age and follow‐up time, were dichotomized. Given the difference in catchment population between sites and that previous studies suggest differences regarding the risk of drug allergy as well as the culprit between adults and children 10, separate regression models for each site were fit.

## Results

**Figure 1:**
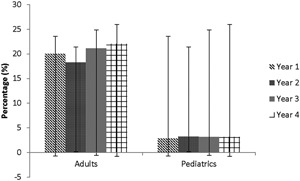
The percentage of drug‐induced anaphylaxis cases among all cases of anaphylaxis per year in the adult and pediatric emergency departments. Error bars represent ± standard deviation.

Temporal trends in the percentage of DIA among all anaphylaxis cases (Table 1 and Fig. 1).

**Table 1:** Percentage and percent difference of anaphylaxis and drug‐induced anaphylaxis cases

Variable (%, 95%CI)	Hôpital Sacré‐Coeur	Montreal Children's Hospital	British Columbia Children's Hospital	Children's Hospital at London Health Science Center
Percentage of anaphylaxis among all ED cases				
2012–2013	0.11% (0.089%, 0.15%)	0.35% (0.31%, 0.40%)	–	–
2013–2014	0.16% (0.13%, 0.20%)	0.33% (0.29%, 0.37%)	–	–
2014–2015	0.15% (0.13%, 0.19%)	0.42% (0.38%, 0.47%)	0.34% (0.29%, 0.40%)	0.097% (0.068%, 0.14%)
2015–2016	0.11% (0.089%, 0.15%)	0.38% (0.34%, 0.42%)	0.40% (0.34%, 0.46%)	0.12% (0.089%, 0.17%)
Differences				
Years 1–2	0.047% (0.019%, 0.091%)	−0.024% (−0.082%, 0.034%)	–	–
Years 2–3	−0.0061% (−0.053%, 0.041%)	0.094% (0.034%, 0.15%)	–	–
Years 3–4	−0.041% (−0.084%, −0.0030%)	−0.046% (−0.11%, 0.017%)	–	–
Total (Years 1–4)	−0.00016% (−0.039%, 0.039%)	0.024% (−0.036%, 0.085%)	0.055% (−0.024%, 0.14%)	0.024% (−0.028%, 0.077%)
Percentage of DIA among all cases of anaphylaxis				
2012–2013	20.0% (11.5%, 32.1%)	2.8% (1.3%, 5.7%)	–	–
2013–2014	18.3% (11.3%, 27.9%)	3.2% (1.6%, 6.3%)	–	–
2014–2015	21.1% (13.5%, 31.2%)	3.5% (1.9%, 6.1%)	2.5% (0.8%, 6.8%)	3.0% (0.16%, 17.5%)
2015–2016	22.1% (13.3%, 34.1%)	3.6% (1.9%, 6.6%)	1.6% (0.41%, 5.0%)	7.0% (1.8%, 20.1%)
Differences				
Year 1–2	−1.7% (−15.5%, 12.1%)	0.4% (−2.8%, 3.6%)	–	–
Year 2–3	2.8% (−9.8%, 15.4%)	0.2% (−2.8%, 3.3%)	–	–
Year 3–4	0.9% (−13.0%, 14.9%)	0.2% (−2.8%, 3.2%)	–	–
Total (Year 1–4)	2.1% (−13.3%, 17.4%)	0.8% (−2.4%, 4.0%)	−0.94% (−4.6%, 2.7%)	0.70% (−16.2%, 8.3%)
Percentage of DIA among all ED visits				
2012–2013	0.023% (0.013%, 0.040%)	0.010% (0.0047%, 0.021%)	–	–
2013–2014	0.029% (0.018%, 0.048%)	0.011% (0.0052%, 0.021%)	–	–
2014–2015	0.033% (0.020%, 0.052%)	0.015% (0.0079%, 0.026%)	0.0086% (0.0028%, 0.024%)	0.0029% (0.0002%, 0.019%)
2015–2016	0.025% (0.015%, 0.043%)	0.014% (0.0072%, 0.025%)	0.0063% (0.0063%, 0.020%)	0.0085% (0.0022%, 0.027%)
Differences				
Years 1–2	0.0065% (−0.014%, 0.027%)	0.00064% (−0.0099%, 0.011%)	–	–
Years 2–3	0.0033% (−0.019%, 0.025%)	0.0039% (−0.0081%, 0.016%)	–	–
Years 3–4	−0.0075% (−0.029%, 0.014%)	−0.00097% (−0.014%, 0.012%)	–	–
Total (Years 1–4)	0.0023% (−0.017%, 0.022%)	0.0036% (−0.083%, 0.016%)	−0.0023% (−0.016%, 0.011%)	0.0055% (−0.0085%, 0.020%)

### Pediatric cases

As shown in Table 1, the percentages of DIA among all cases of anaphylaxis (1.6–7%) and among all ED visits (0.003–0.01%) did not differ and did not change significantly from year to year between the three pediatric EDs. There were no conclusive differences in the percentage of DIA among all cases of anaphylaxis between the three pediatric EDs over 2 years (Table 1).

### Adult cases

At the adult ED, the percentage of DIA among all cases of anaphylaxis (18.3–22.1%) and among all ED visits (0.02–0.03%) also showed no change over a 4‐year period (Table 1). However, the percentage of DIA among all cases of anaphylaxis was substantially higher in the adult center versus the pediatric centers (Table 1).

**Figure 2:**
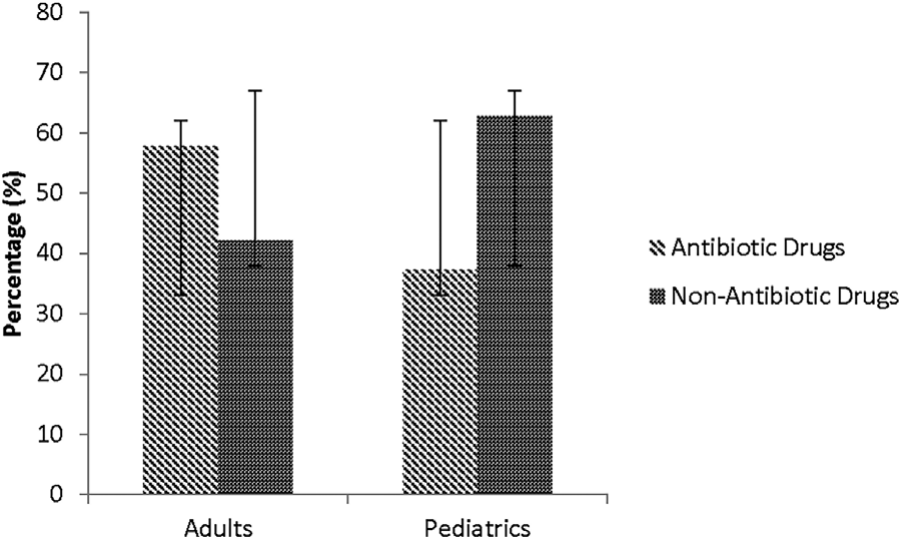
Comparison of the percentage of antibiotic versus non‐antibiotic drug cases among all cases of drug‐induced anaphylaxis cases in the adult and pediatric emergency departments over a 4‐year period. Error bars represent ± standard deviation.

Demographics and clinical characteristics (Table 2 and Fig. 2).

**Table 2:** Characteristics of patients presenting to the emergency department with drug‐induced anaphylaxis

	Adult Patients (*N* = 64)		Pediatric Patients (*N* = 51)		
Variable (%, 95%CI)	No. (%)	95%CI	No. (%)	95%CI	Difference
Age at reaction (median, IQR)	49.4 (40.1, 62.9)		8.00 (3.79, 15.36)		41.4
Age at reaction (mean, standard deviation)	48.9 (14.8)		8.95 (5.9)		39.95 (35.9, 44.0)
Sex (% males)	18 (28.1%)	17.9%, 41.0%	27 (52.9%)	38.6%, 66.8%	−24.8% (−44.2%, −5.5%)
Medication type					
Antibiotics	37 (57.8%)	44.8%, 69.8%	19 (37.3%)	24.5%, 51.9%	20.6% (0.8%, 40.3%)
Beta‐Lactams	18 (28.1%)	17.2%, 41.3%	16 (31.4%)	17.6%, 45.5%	−3.2% (−21.8%, 15.4%)
Macrolides	2 (3.1%)	0%, 16.3%	2 (3.9%)	0%, 18.1%	−0.8% (−8.4%, 6.8%)
Quinolones	13 (20.3%)	9.4%, 33.5%	1 (2.0%)	0%, 16.1%	18.4% (6.0%, 30.7%)
Other antibiotics	4 (6.3%)	0%, 19.4%	0 (0%)	0%, 14.2%	6.3% (−1.4%, 13.9%)
Non‐antibiotic drugs	27 (42.2%)	30.2%, 55.2%	32 (62.7%)	48.1%, 75.5%	−20.6% (−40.3%, −0.8%)
NSAIDs	13 (20.3%)	9.4%, 33.5%	11 (21.6%)	7.8%, 35.7%	−1.3% (−17.5%, 15.0%)
Contrast agents	2 (3.1%)	0%, 16.3%	2 (3.9%)	0%, 18.1%	−0.8% (−8.4%, 3.9%)
Other non‐antibiotic drugs^a^	12 (18.8%)	7.8%, 31.9%	19 (37.3%)	23.5%, 51.4%	−18.5% (−36.6%, ‐0.4%)
Known drug allergy	17 (26.6%)	16.7%, 39.3%	4 (8.3%)	2.7%, 20.9%	18.2% (3.1%, 33.4%)
Known food allergy	8 (12.5%)	5.9%, 23.7%	12 (25.0%)	14.1%, 39.9%	−12.5% (−29.0%, 4.0%)
Known asthma	6 (9.4%)	3.9%, 19.9%	10 (20.8%)	11.0%, 35.4%	−11.5% (−26.8%, 3.9%)
Reaction type					
Mild^b^	0 (0%)	0%, 9.7%	13 (25.5%)	3.7%, 37.3%	−25.5% (−39.2%, −11.8%)
Moderate^c^	53 (82.8%)	75.0%, 92.1%	36 (70.6%)	58.8%, 82.4%	12.2% (−5.1%, 29.5%)
Severe^d^	11 (17.2%)	9.4%, 26.5%	2 (3.9%)	0%, 15.7%	13.3% (0.8%, 25.7%)
Exposure route					
Ingestion	60 (93.8%)	89.1%, 98.7%	38 (74.5%)	64.7%, 86.8%	19.2% (4.1%, 34.4%)
Contact^e^	0 (0%)	0%, 4.9%	3 (5.9%)	0%, 18.2%	−5.9% (−14.1%, 2.3%)
Inhaled	1 (1.6%)	0%, 6.5%	1 (2.0%)	0%, 14.3%	−0.4% (−5.7%, 4.9%)
Parenteral	3 (4.7%)	0%, 9.6%	9 (17.6%)	7.8%, 30.0%	−13.0% (−26.4%, 0.5%)
Treatment in ED					
Epinephrine	33 (51.6%)	38.8%, 64.1%	30 (58.8%)	44.2%, 72.1%	−7.3% (−27.3%, 12.7%)
Antihistamines	53 (82.8%)	70.9%, 90.7%	26 (51.0%)	36.8%, 65.0%	31.8% (13.5%, 50.1%)
Steroids	53 (82.8%)	70.9%, 90.7%	16 (31.4%)	19.5%, 46.0%	51.4% (33.9%, 68.9%)

^a^ Other Non‐Antibiotics Drugs: Children: Marijuana, Local anesthetic (Prilocaine), Antihistamine (Claritin), Corticosteroids (Dexamethasone and Prednisone), N‐acetyl cysteine, Zantac, Oralair, Triptan, Cyclopentolate eye drops, Wilate (Factor 8), Morphine, Vicks VapoDrops, Granulocyte‐macrophage colony‐stimulating factor (GM‐CSF), Atypical Antipsychotic (Risperdal). Adults: Tylenol, Codeine, Cocaine, Alpha1‐Adrenergic Receptor Antagonist (Terazosin), Antifungal Medication (Fluconazole), Lactase (Lacteeze), Benylin cough syrup, Angiotensin‐converting Enzyme (ACE) Inhibitor (Ramipril), Protein Pump Inhibitor (Pantoprazole), Anticonvulsant (Lyrica).

^b^ Symptoms include urticaria, erythema, angioedema, oral pruritus, nausea, nasal congestion, sneezing, rhinorrhea, or throat tightness 11.

^c^ Symptoms include crampy abdominal pain, diarrhea, recurrent vomiting, dyspnea, stridor, cough, wheeze, or “light‐headedness^”^ 11.

^d^ Symptoms include cyanosis, hypoxia, respiratory arrest, hypotension, dysrhythmia, confusion, or loss of consciousness 11.

^e^ Cyclopentolate eye drops.

### Pediatric cases

As shown in Table 2, 51 pediatric patients presented to the three pediatric EDs with DIA. Demographic characteristics (age, sex), presence of co‐morbidities (e.g., asthma), culprit drugs, severity of anaphylaxis, and management were similar in the three pediatric centers. Hence, all pediatric cases were assessed as one group hereafter. Nearly half of the children, 25 patients (49.0%), were recruited prospectively, of which the mean follow‐up time to determine if the patients had been assessed by an allergist was 1.26 years (Supplementary Table S1). The majority of the reactions were triggered by non‐antibiotic drugs (62.7%), of which the main culprit was non‐steroidal anti‐inflammatory drugs (NSAIDs, 21.6%) (Table 2). Reactions attributed to antibiotics accounted for 37.3% of the reactions, with β‐lactams being the most frequently suspected (31.4%). Only four children reported having a history of drug allergy, with one child reacting to the known drug culprit.

### Adult cases

From June 2012 to May 2016, 64 adults presented with DIA of which 52 (81.3%) were recruited prospectively, with a mean follow‐up of 1.33 years (Supplementary Table S1). Unlike pediatric cases, the majority of reactions occurred in females.

Most reactions were attributed to antibiotics (57.8%), mainly β‐lactams (28.1%) and quinolones (20.3%). Reactions attributed to non‐antibiotic drugs accounted for 42.2% of the reactions, NSAIDs being the most frequently involved (20.3%) (Table 2). Seventeen adults reported having a history of drug allergy, of which three had anaphylaxis associated with re‐exposure to a drug they were known to be allergic to. There more severe reactions in adults and a higher percentage had a known drug allergy.

Management in the ED (Table 2).

### Pediatric cases

At the three pediatric centers across Canada, both epinephrine and antihistamines were used to treat over half of the reactions (Table 2). Steroids were used for treatment in 16 (31.4%) patients (Table 2).

### Adult cases

About half of the adults were treated with epinephrine. However, unlike pediatric cases, the majority of cases in adults were treated with antihistamines and/or steroids (Table 2)

**Figure 3:**
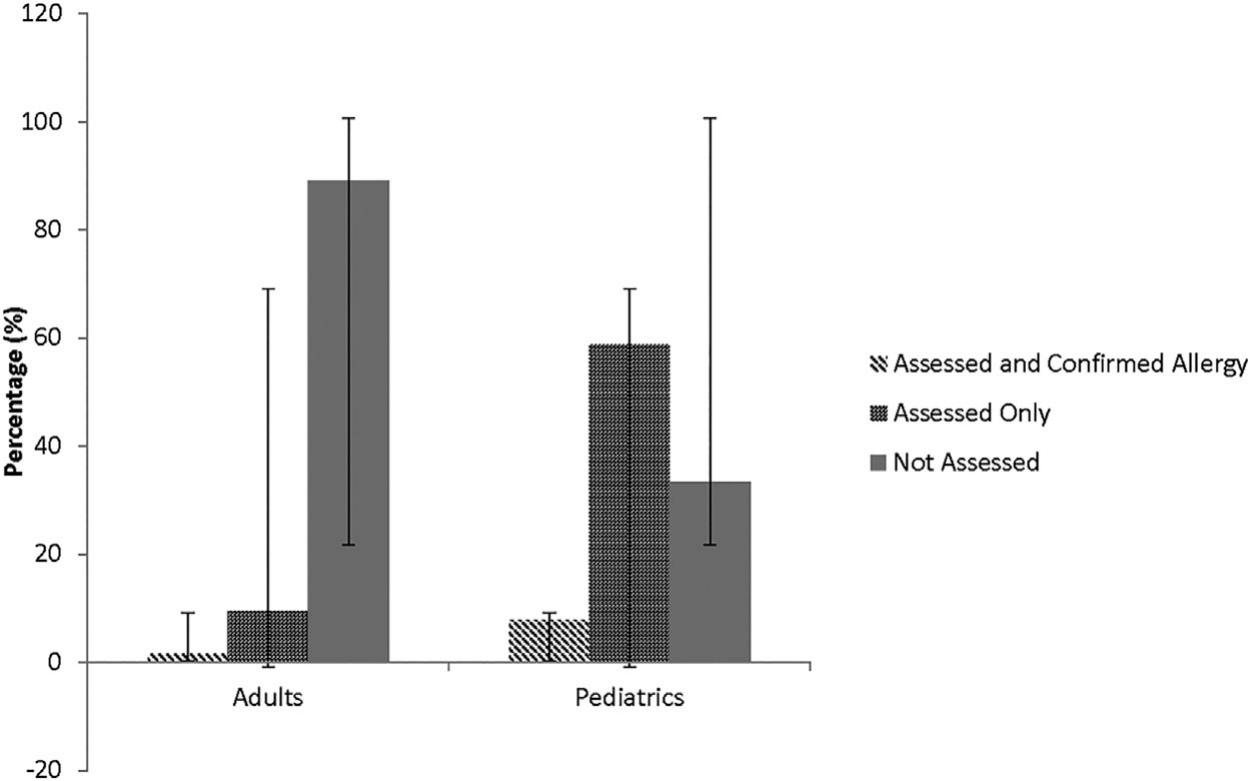
Comparison of the percentage of cases that were assessed and confirmed by an allergist, cases assessed by an allergist only, and cases not assessed among all visits of drug‐induced anaphylaxis in the adult and pediatric emergency departments over a 4‐year period. Error bars represent ± standard deviation.

Allergy assessment (Supplementary Table S2 and Fig. 3).

### Pediatric cases

#### All drugs

Consent for prospective follow‐up was given for 25 pediatric cases of suspected DIA. Data for 20 retrospective cases was collected by chart review of the allergy visits. After the initial ED visit, 30 (68.2%) children had seen an allergist for assessment and medical records were obtained for all children. Fourteen children were skin tested and nine children were subjected to an oral challenge with the suspected drug, either after negative skin test (5, 55.5%), or without prior skin test (4, 44.4%). Drug allergy was diagnosed in 4 (22.2%) of these children, based on positive responses in skin test (2, 14.3%) or oral challenge (2, 22.2%) (Supplementary Table S2).

#### Antibiotics

Of the 18 (40.0%) patients that presented to the pediatric centers with anaphylaxis to antibiotics, only 10 (55.5%) patients had seen an allergist for assessment. Of the 10 children, 7 underwent skin testing, of which 1 was positive to ceftriaxone by intradermal skin testing. Among the six with a negative skin test, two proceeded to a graded oral challenge, which was positive in one case to amoxicillin. Of the three children who did not have a skin test, two underwent an oral challenge without prior skin testing, of which one patient had a positive challenge to clarithromycin. One patient did not undergo any testing despite having seen an allergist.

#### Non‐antibiotics

Among the 27 (60.0%) pediatric patients who reacted to non‐antibiotic drugs, 20 (74.1%) had been assessed by an allergist. Seven patients underwent skin testing of which 6 were negative. The positive skin test was to cyclopentolate. Of the six patients with negative skin tests, three proceeded with a graded oral challenge, which were all negative. Two patients underwent a graded oral challenge without prior skin testing, which were both negative.

### Adult cases

#### All drugs

Among the 64 adult patients with suspected DIA, 52 (81.3%) patients were prospective and eligible for follow‐up. We were able to reach 37 patients (71.2%) of the 52 consenting patients. Less than a third had been assessed by an allergist after the ED visit. Medical charts were obtained for over 50.0% of adults who had seen an allergist (Supplementary Table S2). Of the six (54.5%) adult patients who were assessed by an allergist and provided consent to provide medical records, only two underwent skin testing of which one was reported by the patient as positive to a contrast agent. The patient with the negative skin test had a graded oral challenge which was positive to the antibiotic cefadroxil. Therefore, drug allergy was confirmed by skin test in one patient and an oral challenge in another patient.

#### Antibiotics

Of the 29 (55.8%) adults who had anaphylaxis to antibiotics, 21 (72.4%) consented to follow‐up, of which only 7 (33.3%) had been assessed by an allergist. Of these seven patients, four (57.1%) provided consent to provide medical records. One patient underwent skin testing which was negative and that same patient underwent a graded oral challenge which was positive to cefadroxil.

#### Non‐antibiotics

Of the 23 (44.2%) adult prospective patients who reacted to non‐antibiotic drugs, 16 (69.6%) consented to follow‐up, of which only 3 (18.8%) had been assessed by an allergist. Of these patients, 2 (66.7%) consented to provide medical records. One patient underwent skin testing which was positive to a contrast agent. The second patients did not undergo any testing.

Factors associated with severe DIA, allergy assessment, and diagnosis of DIA (Supplementary Tables S3–S6).

### Pediatric cases

Univariate and multivariate analyses showed that in pediatric patients, parenteral exposure to drugs was the main significant risk factor for severe DIA. Age, sex, type of drug, history of asthma, and/or food allergy were not associated with severe DIA (Supplementary Table S3). Among the patients at the three pediatric centers, assessment by an allergist was more likely in males and in patients presenting to the ED in Montreal versus the other EDs (Supplementary Table S5). An established drug allergy by an allergist through a skin test/challenge was more likely in cases of antibiotic‐induced reactions and less likely in younger children (Supplementary Table S6).

### Adult cases

Similarly, among the adult patients, severe DIA was associated with parenteral exposure when adjusting for age, sex, type of drug, history of asthma, history of known drug allergy, and history of known food allergy (Supplementary Table S4).

## Discussion

We have conducted the first prospective study assessing clinical characteristics and diagnosis of DIA in children and adults in four EDs across Canada. Our study reveals that while there was no conclusive change in the percentage of DIA over time in all four centers, the percentage of DIA among all cases of anaphylaxis is higher in adults than in children. Further, we report the disparities between reported DIA and established DIA in children. The main drug culprits in adults and children are antibiotics and non‐antibiotic drugs, respectively, and, in both age groups, there is substantial underuse of epinephrine. Moreover, our findings show for the first time that the majority of reported cases of DIA cases are not appropriately diagnosed.

The higher percentage of DIA in adults compared to children is consistent with previous retrospective reports suggesting that DIA occurs more frequently in adults 12. The increased risk of DIA in adults could be due to greater exposure to antibiotics over the course of their life and in particular fluoroquinolones, that are relatively contraindicated in children 13. The frequency of use of antibiotic drugs in adults compared to children is further exemplified in Figure 2. Additionally, middle and older aged adults have a greater risk of drug reactions due to the simultaneous use of multiple drugs to treat co‐morbidities and age‐related changes in pharmacokinetics and pharmacodynamics 14. While there was no sex dominance for children, fewer cases of DIA among adult males were found (Table 2), which is in line with other studies 15 and may be explained by the effects of estrogen on mediators of anaphylaxis during the reproductive years in females 15.

In our population, very few adult patients consulted an allergist after the initial ED visit. The low percentage of adults and children assessed for DIA may be due to patient‐related factors or due to the factors related to the Canadian heath system. Studies suggest that young adults, between the ages of 17–44 years, are the least compliant with using referrals to be assessed by medical specialists 16, which could be attributed to patients' other priorities and inability to take time off work 17, 18. Health system‐related factors that could contribute to under assessment of DIA include low number of allergists in Canada and long waiting time for specialist assessment 17. Regardless of its cause, non‐confirmed drug allergy may lead to mislabeling of patients 19. Mislabeling of patients has been associated with increased use of alternative antibiotics 20, 21, 22, increased risk of acquiring antibiotic‐resistant infections 19, 20, 21, 22, such as *C. difficile*, vancomycin‐resistant enterococci (VRE), and methicillin‐resistant Staphylococcus aureus (MRSA), significantly longer hospital stays 19, 21, increased healthcare costs 19, and increased mortality 23.

Our results indicate that majority of suspected DIA cases in adults and children are not assessed appropriately by allergists. Current guidelines recommend the use of skin tests and oral challenges to diagnose suspected cases of antibiotic allergy 24. Studies suggest that skin tests might be useful for diagnosing penicillin allergy 25, however, their role in amoxicillin allergy, the most common penicillin derivative, is less clear 26. Further, skin tests are not standardized for most antibiotics 24 and studies report poor predictive values for antibiotics 24 and NSAIDs 27 regarding skin tests. In the absence of sensitive and accurate skin tests, our results support the use of challenges only to establish the diagnosis of DIA.

An interesting finding is the relatively high percentage of reported fluoroquinolone DIA in adults. Recent studies have found that the number of immediate‐type reactions to quinolones, especially moxifloxacin, increased over the past few years 28, 29, which could be a result of the updated treatment guidelines recommending the use of moxifloxacin as first line treatment in the management of bacterial respiratory infections, including sinusitis and pneumonia in adults 30. Allergy to fluoroquinolone is rarely established likely due to the absence of standardized skin tests 29 and the risks related to conducting a drug challenge 31.

We demonstrate that NSAIDs are a common culprit of DIA in children and adults. NSAIDs were reported to be major triggers of DIA in other studies 32, 33, 34, however none of these studies evaluated the long‐term follow‐up and assessment of those presenting with anaphylaxis to NSAIDs in the ED. The high percentage of reactions to NSAIDs could be explained by the increased consumption and high frequency of prescriptions to treat pain and fever 34, 35. There are no standardized skin tests for the diagnosis of most NSAID‐induced anaphylaxis 32. Recent studies suggest that suspected cases of NSAID allergy should be assessed with oral challenges 24, 27, 36, however, only a few challenges were conducted in our population. The underutilization of challenges in our population is likely attributable to the fact that such challenges are usually only performed in a hospital, under the supervision of an allergist 24 and there is limited access and long wait times for specialist assessment in some areas of Canada 17. It is possible that in cases of DIA attributed to NSAIDS with negative challenges, NSAIDs may have acted as cofactors or augmenting factors rather than as a sole culprit for anaphylaxis 15. It is also possible that cases reported as DIA, with a negative challenge, are likely attributable to the presence of unidentifiable factors or to conditions mimicking anaphylaxis, such as viral infections, food poisoning, or other toxic effects of medications 37.

In our study, the majority of children from the pediatric centers were assessed by an allergist after the initial reaction. Patients recruited from the Montreal pediatric center and males were more likely to be assessed by an allergist. The presence of the large allergy division and a specific drug allergy clinic at the MCH allows for greater access to an allergy specialist compared to the other centers. In addition, given that a large antibiotic registry exists only in the Quebec center and given numerous publications related to this specific registry, there may be higher awareness for referring to allergy specialists at this center 24. Our finding that DIA is more likely established with a skin test and/or challenge in cases of antibiotic‐induced reactions is not surprising given the availability of skin tests for antibiotics (mainly β‐lactams) versus non‐antibiotic drugs 38. It is also possible that in younger children the diagnosis of DIA is less likely established because physicians will be more hesitant to conduct a drug challenge in young children who are less able to verbalize their complaints.

Our study found that receiving parenteral drug treatment was associated with more severe reactions in both adults and children. It is reported that the vast majority of anaphylaxis fatalities have occurred in patients treated with intramuscular or intravenous antibiotic preparations, rather than oral 39, 41. This could be related to receiving a large amount of allergen into the body over a relatively short period of time, which reaches a high concentration in body organs 40. Given the association of a severe reaction with parenteral administration of the drug in children and adults, caregivers should be made aware of the risk for severe anaphylaxis associated with those requiring IV treatment.

Our study has potential limitations. In the case of a negative skin test and negative oral graded challenge, it is possible that cases defined as DIA were actually idiopathic or caused by other unidentified factors. However, this limitation is shared with all studies assessing DIA. Our unique study design allowed for follow‐up of prospective patients and the collection of data on established cases of DIA. Another important limitation is due to the fact that many patients did not see an allergist and many did not have confirmatory tests to establish the cause of suspected DIA. These cases might have been misclassified, however, this is a limitation of all previous studies assessing DIA in the ED. Due to the similarities in terms of medication type, a history of drug allergy, food allergy, and asthma, and severity of reaction between cases that were confirmed and cases that were not confirmed (Tables S7 and S8), this misclassification was unlikely to affect our conclusions. Further, in contrast to other studies on DIA in the ED, our study included a prospective arm that enabled us for the first time to determine the low percentage of established cases and the need for better diagnostic strategies. Given ethics restrictions, we were not allowed to actively assess patients and conduct challenges. Adults were included from one center, since this was the only center collaborating to recruit patients prospectively. Given that the catchment population was based on only four sites across Canada, it is possible that our study cannot be generalized to the entire Canadian pediatric and adult populations. Although we aimed to recruit all patients prospectively, almost 50% of the pediatric patients and 20% of the adult patients were identified retrospectively. Given that we did not have permission to contact retrospective cases, data on assessment of these patients was only available via chart review of the allergy visit. However, demographic and clinical characteristics of DIA between retrospective and prospective patients were similar (Supplementary Table S1) and hence we believe that our findings are valid. Finally, our sample size prevented accurate estimation of the temporal change in percentage of DIA.

In conclusion, this is the first study to assess clinical characteristics and long‐term assessment of DIA presenting in the EDs across Canada. Our study emphasizes the need for uniform guidelines in the management of DIA in the ED such as the regulated use of epinephrine and qualified diagnosis of the condition by trained allergists in order to avoid recurrences and reduce patient morbidity. Future studies elucidating the pathogenesis of DIA and evaluating appropriate and efficient confirmatory tests will contribute to bridging the gaps related to the management of DIA.

## Conflicts of Interest

None.

## Supporting information

Additional supporting information may be found in the online version of this article at the publisher's web‐site.

**Table S1**. Characteristics of Prospective versus Retrospective Patients Presenting to the Emergency Department with Drug‐Induced Anaphylaxis.
**Table S2**. Follow‐up for Diagnosis of Drug Trigger by Allergy Tests.
**Table S3**. Factors Associated with Severe Reactions for All Drugs in Pediatric Patients.
**Table S4**. Factors Associated with Severe Reactions for All Drugs in Adult Patients.
**Table S5**. Factors Associated with Allergy Assessment by and Allergist in Pediatric Patients.
**Table S6**. Factors Associated with an Established Allergy by and Allergist in Pediatric Patients.
**Table S7**. Characteristics of Pediatric Patients assessed by an allergist and underwent testing vs. patients that were not assessed by an allergist.
**Table S8**. Characteristics of Adult Patients assessed by an allergist and underwent testing vs. patients that were not assessed by an allergist.
