# Supplementary material for: Disparities in rate, triggers, and management in pediatric and adult cases of suspected drug‐induced anaphylaxis in Canada
Source: Immun Inflamm Dis. 2017 Nov 1;6(1):3–12. doi: 10.1002/iid3.201 (PMC5818453; doi:10.1002/iid3.201)
Supplement: Supplementary file 1 — Table S1. Characteristics of Prospective versus Retrospective Patients Presenting to the Emergency Department with Drug‐Induced Anaphylaxis. Table S2. Follow‐up for Diagnosis of Drug Trigger by Allergy Tests. Table S3. Factors Associated with Severe Reactions for All Drugs in Pediatric Patients. Table S4. Factors Associated with Severe Reactions for All Drugs in Adult Patients. Table S5. Factors Associated with Allergy Assessment by and Allergist in Pediatric Patients. Table S6. Factors Associated with an Established Allergy by and Allergist in Pediatric Patients. Table S7. Characteristics of Pediatric Patients assessed by an allergist and underwent testing vs. patients that were not assessed by an allergist. Table S8. Characteristics of Adult Patients assessed by an allergist and underwent testing vs. patients that were not assessed by an allergist. [file IID3-6-3-s001.docx]

**Table S1. Characteristics of Prospective versus Retrospective Patients Presenting to the Emergency Department with Drug-Induced Anaphylaxis**

| **Variable (%, 95%CI)** | **Adult Patients** | | | **Pediatric Patients** | | |
| --- | --- | --- | --- | --- | --- | --- |
|  | **Prospective (N=52)** | **Retrospective (N=12)** | **Difference** | **Prospective (N=25)** | **Retrospective (N=26)** | **Difference** |
| Age at Reaction (mean, standard deviation) | 49.3 (14.0) | 47.1 (18.3) | 2.2 (-9.9, 14.3) | 8.7 (6.3) | 9.2 (5.7) | -0.5 (-3.8, 2.9) |
| Length of follow-up in years (mean, standard deviation) | 1.33 (0.40) |  |  | 1.26 (0.46) |  |  |
| Sex (% males) | 30.8% (19.1%, 45.3%) | 16.7% (2.9%, 49.1%) | 14.1% (-15.6%, 43.8%) | 64.0% (42.6%, 81.3%) | 42.3% (24.0%, 62.8%) | 21.7% (-9.0%, 52.3%) |
| **Medication Type**  Antibiotics  Non-Antibiotic Drugs | 55.8% (41.4%, 69.3%)  44.2% (30.7%, 58.6%) | 66.7% (35.4%, 88.7%)  33.3% (11.3%, 64.6%) | -10.9% (-45.9%, 24.1%)  10.9% (-24.1%, 45.9%) | 32.0% (15.7%, 53.6%)  68.0% (46.4%, 84.3%) | 42.3% (24.0%, 62.8%)  57.7% (37.2%, 76.0%) | -10.3% (-40.6%, 20.0%)  10.3% (-20.0%, 40.6%) |
| Known Drug Allergy | 26.9% (16.0%, 41.3%) | 25.0% (6.7%, 57.2%) | 1.9% (-27.3%, 31.2%) | 4.0% (0.2%, 22.3%) | 13.0% (3.4%, 34.7%) | -3.7% (-20.2%, 12.8%) |
| Known Food Allergy | 13.5% (6.0%, 26.4%) | 8.3% (0.4%, 40.2%) | 5.1% (-18.2%, 28.4%) | 40.0% (21.8%, 61.1%) | 8.7% (1.5%, 29.5%) | 32.3% (6.6%, 58.0%) |
| Known Asthma | 7.7% (2.5%, 19.4%) | 16.7%( 2.9%, 49.1%) | -9.0% (-36.4%, 18.4%) | 32.0% (15.7%, 53.6%) | 8.7% (1.5%, 29.5%) | 24.3% (-0.6%, 49.2%) |
| **Reaction type**  Mild^a^  Moderate^b^  Severe^c^ | 0% (0%, 11.0%)  82.7% (75.0%, 93.7%)  17.3% (9.6%, 28.3%) | 0% (0%, 24.2%)  83.3% (75.0%, 100%)  16.7% (8.3%, 40.9%) | 0% (0%, 0%)  -0.6% (-24.7%, 23.5%)  -0.6% (-23.5%, 24.7%) | 24.0% (8.0%, 41.6%)  68.0% (52.0%, 85.6%)  8.0% (0%, 25.6%) | 26.9%(11.5%, 42.3%)  73.1% (57.7%, 88.5%)  0% (0%, 15.4%) | -2.9% (-29.7%, 23.9)  -5.1% (-34.0%, 23.8%)  8.0% (-6.6%, 22.6%) |
| **Exposure Route**  Ingestion  Contact^d^  Inhaled  Parenteral | 91.8% (85.7%, 98.2%)  0% (0%, 6.3%)  2.0% (0%, 8.4%)  6.1% (0%, 12.4%) | 100% (100%, 100%)  0% (0%, 13.5%)  0% (0%, 13.5%)  0% (0%, 13.5%) | -8.2% (-21.0%, 4.7%)  0% (0%, 0%)  2.0% (-4.0%, 8.0%)  6.1% (-5.8%, 18.0%) | 68.0% (52.0%, 85.3%)  4.0% (0%, 21.3%)  4.0% (0%, 21.3%)  24.0% (8.0%, 41.3%) | 80.8% (69.2%, 95.5%)  7.7% (0%, 22.5%)  0% (0%, 14.8%)  11.5% (0%, 26.3%) | -12.8% (-40.4%, 14.9%)  -3.7% (-20.2%, 12.8%)  4.0% (-7.6%, 15.6%)  12.5% (-12.2%, 11.5%) |
| **Treatment in ED**  Epinephrine  Antihistamines  Steroids | 55.8% (41.4%, 69.3%)  86.5% (73.6%, 94.0%)  80.8% (67.0%, 89.9%) | 33.3% (11.3%, 64.6%)  66.7% (35.4%, 88.7%)  91.7% (59.8%, 99.6%) | 22.4% (-12.6%, 57.5%)  19.9% (-13.5%, 53.2%)  -10.9% (-35.0%, 13.2%) | 80.0% (58.7%, 92.4%)  48.0% (28.3%, 68.2%)  32.0% (15.7%, 53.6%) | 38.5% (20.9%, 59.3%)  53.8% (33.7%, 72.9%)  30.8% (15.1%, 51.9%) | 40.0% (13.2%, 69.9%)  -5.8% (-37.2%, 25.5%)  -29.5% (-59.6%, 0.5%) |

^a^Symptoms include urticaria, erythema, angioedema, oral pruritus, nausea, nasal congestion, sneezing, rhinorrhea or throat tightness^(12)^

^b^Symptoms include crampy abdominal pain, diarrhea, recurrent vomiting, dyspnea, stridor, cough, wheeze, or “light-headedness^”(12)^

^c^Symptoms include cyanosis, hypoxia, respiratory arrest, hypotension, dysrhythmia, confusion, or loss of consciousness^.(12)^

^d^Cyclopentolate eye drops

**Table S2. Follow-up for Diagnosis of Drug Trigger by Allergy Tests**

| **Variable (%, 95%CI)** | **Pediatric Patients (N=45)** | **Adult Patients**  **(N=52)** | **Difference** |
| --- | --- | --- | --- |
| Responded to follow up | 44 (97.8%) | 37 (71.2%) | 8 (26.6%) |
| Saw Allergist | 68.2% (52.3%, 80.9%) | 29.7%, (16.4%, 47.2%) | 36.9% (14.3%, 59.6%) |
| Consented to Provide Medical Record | 100% (85.9%, 100%) | 54.5% (24.67%, 81.9%) | 45.5% (9.8%, 81.1%) |
| **Skin Test Only** | 30.0% (13.3%, 49.0%) | 16.7% (0%, 62.0%) | 13.3% (-30.7%, 57.4%) |
| **Oral Challenge Only** | 13.3% (0%, 32.4%) | 0% (0%, 45.3%) | 13.3% (-8.8%, 35.5%) |
| **Skin Test and Oral Challenge** | 16.7% (0%, 35.7%) | 16.7% (0%, 62.0%) | 0% (-32.7%, 32.7%) |
| No Tests | 40.0% (23.3%, 59.0%) | 66.7% (50.0%, 100%) | -26.7% (-78.3%, 24.9%) |
| Diagnosed by Skin Test | 14.3% (2.5%, 43.8%) | 50.0% (9.5%, 90.5%) | -35.7% (-100%, 64.5%) |
| Diagnosed by Oral Challenge | 22.2% (3.9%, 59.8%) | 100% (5.5%, 100%) | -77.8% (-100%, 4.9%) |
| Established drug allergy by Skin test/Challenge in those assessed by allergist | 13.3% (4.4%, 31.6%) | 33.3% (6.0%, 75.9%) | -20.0% (-69.6%, 29.6%) |
| Positive tests among all those that underwent skin test/challenge | 22.2% (7.4%, 48.1%) | 100% (19.8%, 100%) | -77.8% (-100%, -30.8%) |

**Table S3. Factors Associated with Severe Reactions for All Drugs in Pediatric Patients**

| Pediatric Patients (N=51) | | |
| --- | --- | --- |
|  | **Univariate** | **Multivariate^a^** |
| Characteristics | OR (95% CI) | OR (95% CI) |
| Age at reaction | 1.00 (0.99, 1.01) | 1.00 (0.99, 1.01) |
| Sex (Males) | 1.08 (0.97, 1.20) | 1.09 (0.97, 1.22) |
| Antibiotics | 1.02 (0.91, 1.14) | 1.02 (0.91, 1.15) |
| Known Asthma | 0.95 (0.82, 1.09) | 0.97 (0.84, 1.13) |
| Known Drug Allergy | 0.96 (0.78, 1.18) | 0.94 (0.77, 1.15) |
| Known Food Allergy | 0.95 (0.83, 1.08) | 0.96 (0.84, 1.11) |
| Parenteral Exposure | 1.25 (1.10, 1.42) | 1.23 (1.07, 1.43) |

^a^Adjusted Odds Ratio

**Table S4. Factors Associated with Severe Reactions for All Drugs in Adult Patients**

| Adult Patients (N=64) | | |
| --- | --- | --- |
|  | **Univariate** | **Multivariate^a^** |
| Characteristics | OR (95% CI) | OR (95% CI) |
| Age at reaction | 0.99 (0.99, 1.01) | 0.99 (0.99, 1.00) |
| Sex (Males) | 0.85 (0.69, 1.04) | 0.83 (0.66, 1.03) |
| Antibiotic | 0.92 (0.76, 1.11) | 0.96 (0.78, 1.17) |
| Known Asthma | 0.99 (0.72, 1.37) | 1.03 (0.72, 1.47) |
| Known Drug Allergy | 1.01 (0.81, 1.24) | 0.93 (0.74, 1.17) |
| Known Food Allergy | 1.09 (0.82, 1.45) | 0.97 (0.71, 1.31) |
| Parenteral Exposure | 1.67 (1.08, 2.58) | 1.76 (1.10, 2.80) |

^a^Adjusted Odds Ratios

**Table S5. Factors Associated with Allergy Assessment by and Allergist in Pediatric Patients**

| Pediatric Patients (N=51) | | |
| --- | --- | --- |
|  | **Univariate** | **Multivariate^a^** |
| Characteristics | OR (95% CI) | OR (95% CI) |
| Age at reaction | 0.99 (0.97, 1.01) | 0.99 (0.97, 1.01) |
| Sex (Males) | 1.13 (0.86, 1.47) | 1.32 (1.01, 1.73) |
| Antibiotics | 0.92 (0.69, 1.22) | 1.11 (0.84, 1.46) |
| Quebec Centre | 1.78 (1.19, 2.68) | 1.82 (1.10, 3.01) |
| Parenteral Exposure | 0.83 (0.60, 1.16) | 0.98 (0.68, 1.42) |
| Severe Reaction | 0.75 (0.41, 1.38) | 0.57 (0.29, 1.11) |
| Epinephrine Treatment | 1.06 (0.80, 1.40) | 1.16 (0.89, 1.51) |

^a^Adjusted Odds Ratios

**Table S6. Factors Associated with an Established Allergy by and Allergist in Pediatric Patients**

| Pediatric Patients (N=51) | | |
| --- | --- | --- |
|  | **Univariate** | **Multivariate^a^** |
| Characteristics | OR (95% CI) | OR (95% CI) |
| Age at reaction | 0.98 (0.96, 1.00) | 0.98 (0.96, 0.99) |
| Sex (Males) | 0.77 (0.61, 0.97) | 0.82 (0.66, 1.02) |
| Antibiotics | 1.28 (0.99, 1.65) | 1.34 (1.05, 1.71) |
| Quebec Centre | 1.15 (0.57, 2.31) | 0.97 (0.47, 2.04) |
| Parenteral Exposure | 1.08 (0.77, 1.52) | 1.12 (0.78, 1.62) |
| Severe Reaction | 0.87 (0.43, 1.75) | 0.76 (0.37, 1.56) |
| Epinephrine Treatment | 1.23 (0.96, 1.58) | 1.24 (0.99, 1.56) |

^a^Adjusted Odds Ratios

**Table S7. Characteristics of Pediatric Patients assessed by an allergist and underwent testing vs. patients that were not assessed by an allergist**

|  | **Assessment and Testing (n=17)** | | **No Assessment or No Testing (n=23)** | | **Difference** |
| --- | --- | --- | --- | --- | --- |
| **Variable** | **No. (%)** | **95%CI** | **No. (%)** | **95% CI** |  |
| Age at Reaction (median, IQR) | 9.7 (6.5, 15.6) |  | 6.2 (3.8, 15.5) |  | 3.5 |
| Age at Reaction (mean, standard deviation) | 9.8 (6.2) |  | 8.7 (6.1) |  | 1.1 |
| Sex (% males) | 7 (41) | 9.4%, 66.5% | 13 (57) | 34.9%, 76.1% | -15.3% (-51.4%, 20.7%) |
| **Medication Type** |  |  |  |  |  |
| Antibiotics | 9 (53) | 28.5%, 76.1% | 7 (30) | 14.1%, 53.0% | 22.5% (-12.9%, 57.9%) |
| Non-Antibiotic Drugs | 8 (47) | 23.9%, 71.5% | 16 (70) | 47.0%, 85.9% | -22.5% (-57.9%, 12.9%) |
| Known Drug Allergy | 0 (0) | 0%, 22.9% | 3 (13) | 3.4%, 34.7% | -13.0% (-31.9%, 5.8%) |
| Known Food Allergy | 4 (24) | 7.8%, 50.2% | 6 (26) | 11.1%, 48.7% | -2.6% (-32.1%, 27.0%) |
| Known Asthma | 5 (29) | 11.4%, 56.0% | 5 (22) | 8.3%, 44.2% | 7.7% (-24.9%, 40.2%) |
| **Reaction Type** |  |  |  |  |  |
| Mild Reaction | 2 (12) | 0%, 30.4% | 6 (26) | 13.0%, 47.1% | -14.3% (-43.0%, 14.4%) |
| Moderate Reaction | 14 (82) | 70.6%, 100% | 16 (70) | 56.5%, 90.6% | 12.8% (-18.4%, 44.0%) |
| Severe Reaction | 1 (6) | 0%, 24.6% | 1 (4) | 0%, 25.4% | 1.5% (-13.9%, 4.3%) |

**Table S8. Characteristics of Adult Patients assessed by an allergist and underwent testing vs. patients that were not assessed by an allergist**

|  | **Assessment and Testing (n=2)** | | **No Assessment or No Testing (n=62)** | | **Difference** |
| --- | --- | --- | --- | --- | --- |
| **Variable** | **No. (%)** | **95%CI** | **No. (%)** | **95% CI** |  |
| Age at Reaction (median, IQR) | 70.9 (70.8, 70.9) |  | 49.1 (39.8, 61.9) |  | 21.8 |
| Age at Reaction (mean, standard deviation) | 70.9 (0.15) |  | 48.2 (14.7) |  | 22.7 |
| Sex (% males) | 1 (50) | 9.5%, 90.5% | 17 (27) | 17.2%, 40.4% | 22.6% (-70.2%, 100%) |
| **Medication Type** |  |  |  |  |  |
| Antibiotics | 1 (50) | 9.5%, 90.5% | 36 (58) | 44.9%, 70.3% | -8.1% (-86.5%, 70.4%) |
| Non-Antibiotic Drugs | 1 (50) | 9.5%, 90.5% | 26 (42) | 29.7%, 55.1% | 8.1% (-70.4%, 86.5%) |
| Known Drug Allergy | 1 (50) | 9.5%, 90.5% | 16 (26) | 15.9%, 38.7% | 24.2% (-70.1%, 100%) |
| Known Food Allergy | 1 (50) | 9.5%, 90.5% | 7 (11) | 5.0%, 22.5% | 38.7% (-56.8%, 100%) |
| Known Asthma | 0 (0) | 0%, 80.2% | 6 (10) | 4.0%, 20.5% | -9.7% (-26.7%, 7.4%) |
| **Reaction Type** |  |  |  |  |  |
| Mild Reaction | 0 (0) | 0%, 95.8% | 0 (0) | 0%, 8.7% | 0% (0%, 0%) |
| Moderate Reaction | 1 (50) | 50%, 100% | 52 (84) | 75.8%, 92.5% | -33.9% (-100%, 61.8%) |
| Severe Reaction | 1 (50) | 50%, 100% | 10 (16) | 8.1%, 24.8% | 33.9% (-61.8%, 100%) |
